# The Laschamp geomagnetic excursion featured in nitrate record from EPICA-Dome C ice core

**DOI:** 10.1038/srep20235

**Published:** 2016-01-28

**Authors:** R. Traversi, S. Becagli, S. Poluianov, M. Severi, S. K. Solanki, I. G. Usoskin, R. Udisti

**Affiliations:** 1 Department of Chemistry “Ugo Schiff”, University of Florence, Via della Lastruccia, 3 I-50019, Sesto F.no (Florence), Italy; 2 ReSoLVE Center of Excellence, Faculty of Sciences, University of Oulu, PL 3000, FIN-90014 Finland; 3 Max-Planck-Institut für Sonnensystemforschung, Justus-von-Liebig-Weg 3, D-37077, Göttingen, Germany; 4 School of Space Research, Kyung Hee University, Yongin, Gyeonggi, 446-701, South Korea; 5 Sodankylä Geophysical Observatory, Oulu unit, FIN-90014 University of Oulu, Finland

## Abstract

Here we present the first direct comparison of cosmogenic ^10^Be and chemical species in the period of 38–45.5 kyr BP spanning the Laschamp geomagnetic excursion from the EPICA-Dome C ice core. A principal component analysis (PCA) allowed to group different components as a function of the main sources, transport and deposition processes affecting the atmospheric aerosol at Dome C. Moreover, a wavelet analysis highlighted the high coherence and in-phase relationship between ^10^Be and nitrate at this time. The evident preferential association of ^10^Be with nitrate rather than with other chemical species was ascribed to the presence of a distinct source, here labelled as “cosmogenic”. Both the PCA and wavelet analyses ruled out a significant role of calcium in driving the ^10^Be and nitrate relationship, which is particularly relevant for a plateau site such as Dome C, especially in the glacial period during which the Laschamp excursion took place. The evidence that the nitrate record from the EDC ice core is able to capture the Laschamp event hints toward the possibility of using this marker for studying galactic cosmic ray flux variations and thus also major geomagnetic field excursions at pluri-centennial-millennial time scales, thus opening up new perspectives in paleoclimatic studies.

Archival indicators of cosmic ray flux are particularly important, since this flux is affected by a number of important variables such as the geomagnetic field, the heliomagnetic field and possibly the local interstellar medium. So far the main such indicators are cosmogenic radionuclides ^10^Be and ^14^C, which allow this flux to be followed over the Holocene. Each of these has its limitations, so that additional proxies of the cosmic ray flux are strongly pursued. Nitrate in polar ice cores has been proposed as such an indicator/proxy, but its validity is still under debate. Here we employ a prominent anomaly seen in ^10^Be during the last glacial, the Laschamp event due to a geomagnetic excursion, to test whether nitrate also displays signs of anomalously high cosmic ray flux.

The Laschamp excursion stands out as the most intense geomagnetic event occurring over the last 50 kyr, with a quasi-reversed polarity of the geomagnetic field^1^.

The event has been dated in marine cores and volcanic rocks to lie within the range 39–41 kyr BP^2^,^3^,^4^. The Laschamp excursion was highlighted in different natural archives from both hemispheres as a dramatic paleointensity drop in lava flows^4^,^5^ and sediment cores^6^,^7^,^8^,^9^ demonstrating the global nature distribution of the geomagnetic anomaly.

Already since the late 1980′s also strong enhancements of ^10^Be content observed in both Arctic and Antarctic ice^10^,^11^,^12^,^13^,^14^,^15^ and sediments^16^ were related to geomagnetic field excursions.

Indeed, the weakened geomagnetic field intensity leads to a less effective shield for the penetration of cosmic rays into the Earth’s atmosphere leading to an increased production of cosmogenic isotopes, such as ^10^Be. The production of ^10^Be is directly related to the intensity of the cosmic ray flux on the Earth’s upper atmosphere, which is determined by the galactic cosmic ray flux outside the heliosphere and by modulation by the heliomagnetic and geomagnetic fields^17^,^18^. Once formed by spallation of oxygen and nitrogen in the atmosphere by cosmic rays, ^10^Be is quickly attached to aerosols and removed by precipitation to the surface of the Earth^17^.

Although the cosmic ray flux in the geomagnetic polar regions may be naively expected to be insensitive to changes in the dipole moment intensity (since the geomagnetic field lines are open at the geomagnetic poles), simulations by general circulation models^19^,^20^ show that ^10^Be is partly mixed in the atmosphere, leading to a clear dependence on the geomagnetic field strength. The ^10^Be depositions in polar areas should vary by roughly a factor of two between the normal (the last century) and low (like during the Laschamp event) geomagnetic fields^21^. It is assumed that ^10^Be is redistributed, after local production, by atmospheric dynamics on a global scale, as demonstrated by the excellent agreement of several authigenic ^10^Be/^9^Be records obtained from sediments with the ^10^Be flux record of Greenland ice cores^22^. The validity of the GCM model to reproduce atmospheric transport of Be isotopes was proven by studies on shorter time scales^23^.

Basing on the available ^10^Be records from marine and ice cores it is now generally agreed that the ^10^Be peak at about 41 kyr BP is a production impulse resulting from the geomagnetic dipole minimum (the Laschamp excursion)^15^.

Such a correspondence has been and is still particularly relevant for ice core dating purposes. For instance, the ^10^Be peak corresponding to the Laschamp event from the EPICA Dome C ice core was used as a tie point in the recent AICC12 dating, synchronizing EDC with NGRIP ice core^24^,^25^.

Hence, so far the Laschamp event has been identified in sediment cores and volcanic rocks by paleomagnetism studies and in deep polar ice cores by ^10^Be measurements. Here we show for the first time that the nitrate record from the EPICA Dome C ice core also displays an excursion during the Laschamp event, in agreement with ^10^Be record from the same ice core.

The origin of nitrate in the Antarctic atmosphere is not fully understood and the cosmogenic source of nitrate in polar ice cores is currently an object of discussion since it arises by a combination of inputs from the stratosphere, from low latitude sources, and recycling from snowpack through photolysis and evaporation/condensation processes^26^,^27^,^28^.

Unfortunately, due to such hindrances, at now there is no readily available model able to address properly nitrate sources, transport and deposition in polar areas, neither in present nor in glacial time, and presently we have to rely on available experimental observations. Although a precise quantification of nitrate sources has not been yet achieved, the analysis of N and O isotope composition in nitrate in Antarctic aerosol and snow has been used to find a fingerprint of the different nitrate inputs^29^,^30^.

Isotope analysis on aerosol collected at Dome C has shown the dominance of a stratospheric source in specific times of year, namely in late austral winter-early spring, through Polar Stratospheric Clouds sedimentation, whereas re-emission from the snowpack appears to be the most relevant contribution in full summer^26^. By using a similar approach, Michalski *et al.*^31^ suggest that nitrate in Antarctic atmosphere is the result of a mixing of stratospheric and tropospheric sources, roughly in equal amount along the year. Notwithstanding, the relative importance of stratospheric and tropospheric inputs to Antarctic nitrate is still an unsolved issue.

As concerning the possible influence of GCR on tropospheric nitrate concentration, a recent model study^32^ computed enhancements of 15% in nitrate precursors (NOy) content for the Antarctic stratosphere just during the Laschamp event, due to an increased GCR-induced ionization. Other model studies were devoted to the investigation of the influence of GCR on atmospheric composition, including NOx, in current times. Semeniuk *et al.*^33^ used CMAM (Canadian Middle Atmosphere Model), which is a chemistry climate model, customized in order to take into account solar irradiance cycle, and found a GCR-induced increase in NOy up to about 40% in the troposphere. Also Calisto *et al.*^34^, by using the 3-D Chemistry Climate Model (CCM) SOCOL v2.0, observed statistically significant effects of GCR on tropospheric and stratospheric NOx. In particular, for the Southern Hemisphere, SOCOL model suggests that the GCR-induced NOx increase exceeds 10% in the tropopause region, reaching 20% at the pole.

Moreover, the occurrence of intense nitrate inputs from the stratosphere at Dome C was revealed by the analysis of nitrate content in current atmospheric aerosol^35^. Also, nitrogen isotopic composition studies highlighted the possibility of such inputs in specific time periods along the year^29^,^30^, but a nitrate source apportionment for this site, or for the central Antarctic plateau is far from being achieved to date.

Due to multiple sources of nitrate and possible post-depositional effects, the possibility of recovering a stratospheric (and hence possibly cosmogenic) fingerprint from nitrate in Antarctic ice cores is still open and appears so far to be highly dependent on the features of the drilling site (mostly geographical location and accumulation rate) and on the analysis resolution of the ice core. For example, the detection of impulsive nitrate events caused by Solar Particle Events (SPEs) in polar ice cores is heavily debated^36^,^37^,^38^. However, the present study is not related to such short-term spikes.

Concerning longer-term relationships of polar nitrate with solar activity, another Antarctic ice core (TALDICE) showed a significant relationship between nitrate record and cosmogenic isotopes (^10^Be and ^14^C) at the multi-centennial-millennial scale in the Holocene and such a relationship was ascribed to a common cosmogenic source and stratospheric transport^39^,^40^. The same nitrate record was used, together with other worldwide distributed records, to relate solar activity variations to climate variability^41^.

On the basis of these results, it seems worthwhile to investigate thoroughly a possible relationship of nitrate records from polar ice with the record of a cosmogenic isotope measured on the same ice core. The simultaneous availability of ^10^Be and nitrate data from the Laschamp event in the EPICA Dome C ice core made it possible to compare these two records, together with the other chemical parameters determined along with nitrate with sufficient reliability (Na^+^, Cl^−^, K^+^, Mg^2+^, Ca^2+^, SO_4_^2−^, MSA), without invoking possible dating mismatch. Here we show that the nitrate record shows an association with ^10^Be during the period of the Laschamp event unlike the rest of the measured chemical markers confirming the ability of this marker to catch major, relatively long-term (millennial scale) changes in cosmic ray flux impinging on the Earth.

## Methods

^10^Be and ion chemistry data presented here are relative to the EPICA-Dome C ice core (EDC henceforth), drilled in the framework of the European Project for Ice Coring in Antarctica (EPICA), at Dome C (East Antarctica, 75° 06′ S, 123° 21′ E, altitude 3233 m above sea level). Being located on the ice sheet at high elevation and about 1100 km from the coastline, this site is not significantly affected by local terrestrial sources and “internal” sea areas (e.g. Ross Sea) and therefore it can be considered representative of mainly global inputs and atmospheric circulation.

The data used in this paper belong to the 697.96–787.56 m depth range of the EDC ice core.

As concerning the ion chemistry, the samples were collected into sample vials using a melting device, as accomplished for all the ice strips below 580 m depth. A strip (3.4 × 3.4 cm, 55 cm long) of ice was melted onto a hotplate in the field^42^,^43^, and part of the melt from the inner part of the core was fed into vials for later ion chromatography (IC) analysis; along the entire core another part of the melt was led directly to various detection devices in a continuous flow analysis-CFA system. For nitrate, sulphate and chloride, the depth range here investigated was analysed both by conventional Ion Chromatography as well as passing some of the CFA water directly into a fast ion chromatography (FIC) device^44^,^45^. A comparison of the two latter methods shows excellent agreement^45^, but due to the capacity of the conventional IC of carrying out also the measurement of the mentioned chemical parameters, we chose to use only the whole discrete IC data set and to leave aside the FIC data, to rule out possible methodological biases.

The ion data presented here were measured in different European laboratories; the estimated uncertainty on individual measurements is better than 5%^46^ during glacial periods like the one under investigation, when larger concentrations of chemical markers are measured, while it increases during interglacial periods when measured levels decrease.

The ^10^Be measurements were carried out at the Gif-sur-Yvette Tandetron based AMS facility. The 55 cm long ice strips were divided into 5 samples of 11 cm in length, each representing about 9 years. The used analytical procedure was modified with respect to previous works^11^,^13^, due to the lower amount of ice per sample (about 50 g, i.e. almost an order of magnitude smaller) and details are reported by Raisbeck *et al.*^47^. The overall measurement uncertainty was estimated to be about 8.5%.

Considering that ^10^Be and nitrate are mainly removed from the atmosphere through dry deposition^12^,^39^,^48^ at this site, an appropriate parameter to study production variations is the deposition flux, i.e. the product of the measured concentrations and the estimated accumulation rates. The used time scale for dating the IC and ^10^Be samples is AICC12^24^,^25^, which also provides the accumulation rate values used here for calculating the deposition flux.

Given the different analysis resolution of the samples analysed for chemistry (ranging between 6 and 37 cm with an average value of 13 cm, due to the above described sub-sampling method) and ^10^Be (constant 11 cm resolution) and the lack of a correspondence of the samples analysed for chemistry and ^10^Be, the latter were resampled in order to match the data set with the lowest resolution, by using the age values returned by the cubic spline function applied to the age vs. depth curve of the IC data.

To investigate the data sets we applied the principal component analysis (PCA henceforth) and the wavelet coherence analysis. For PCA the data were standardized, i.e., their mean and standard deviation were set to zero and one, respectively.

PCA extracts common factors from a data set with corresponding loadings indicating how important a particular factor is for description of a given series. The analysis can disentangle independent processes that may commonly drive different variables. In this work PCA is provided by the commercial software StatSoft STATISTICA 7.0. The analysis was done with the standard PCA transformation and consequent rotation of factor loadings according to the “varimax” criterion^49^.

The wavelet coherence analysis examines covariability between two data series^50^. It is a generalization of the correlation analysis in time and frequency domains. The method uses the wavelet spectral transformation instead of the classical Fourier and allows to study more realistic non-stationary signals. In this work the analysis was realized with a free-distributed MATLAB code written by Grinsted *et al.*^51^, but containing a notable modification in the procedure of the significance estimate of results. For the significance test we used the random-phase non-parametric method proposed by Ebisuzaki^52^, which is more conservative and physically reasonable, than the default red-noise test^39^,^53^. Additionally, for the significance estimate we have included also the coherence factor |cos θ| taking the relative phase between the series θ into account. The wavelet transformation had the base Morlet-wavelet with the parameter ω_0_ = 3. The estimation of the significance was done for the level of 5% according to the Monte-Carlo algorithm with 1000 iterations.

## Results and Discussion

**Figure 1:**
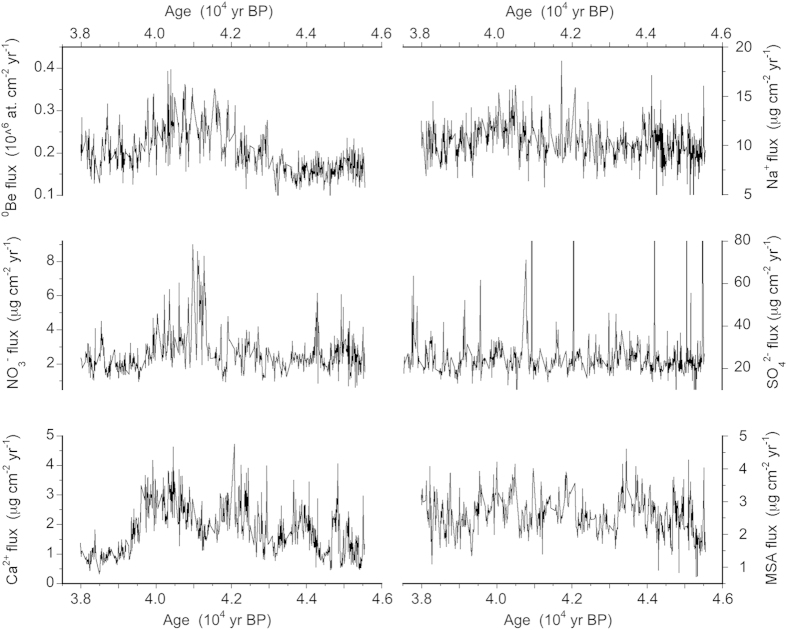
Flux temporal profiles for ^10^Be and the most relevant chemical markers measured along the EDC ice core in the 38–45.5 kyr BP time period.

Figure 1 shows the ^10^Be and ions flux data from the EDC ice core in the period of time related to the Laschamp event.

Considering that the dominant mechanism of atmospheric removal is at Dome C dry deposition^48^,^53^, especially in the glacial period during which the Laschamp excursion took place, we chose to report fluxes instead of the original concentration data, in order to rule out the possible role of accumulation rate in modulating the concentration of chemical species in the snow layers.

As shown in Fig. 1 and reported in detail by Raisbeck *et al.*^47^, the ^10^Be peak is centred at 41 kyr BP (740 m depth) and spans about 2500 kyr.

The ionic markers (NO_3_^−^, Ca^2+^, Na^+^, SO_4_^2−^, MSA) shown in the same figure exhibit different patterns in this specific period. One can see that SO_4_^2−^ shows high abrupt spikes, related to volcanic eruptions, superimposed to a constant background, as often found in EDC ice core (e.g. 58). This behaviour does not appear to be associated with ^10^Be variability according to a visual inspection. Similarly, the MSA record displays a larger variability of background values, but without evident spikes in flux, befitting the gradual variations of biogenic source, as observed all along the EDC ice core^54^. However, no resemblance with the ^10^Be pattern is visible. A different (centennial-millennial) variability is featured by Na^+^ and Ca^2+^, showing larger peaks (500–2000 yr long), usually in phase between them but not in phase with the main one exhibited by ^10^Be. Unlike these markers, nitrate shows a similar pattern to ^10^Be, with a major enhancement between 39.5 and 42.5 kyr BP, with peak values up to 4 times higher than the background. Note, however, that ^10^Be shows a broad peak with relatively constant “peak” concentration, whereas nitrate exhibits a progressive increase of the levels from 42.5 to 41 kyr BP, with the peak values lasting about 500 years (against the 2000 yr for ^10^Be).

Since the simple visual comparison of the deposition flux data can provide only a hint to a common behaviour of ^10^Be and nitrate during the Laschamp event, we applied a Principal Component Analysis to all the available measured parameters, in order to assess the existence and the extent of a common variability for ^10^Be and the other chemical parameters.

Table 1 reports the obtained results with indication of factor loadings. Four factors were identified, explaining about 80% of the variance of the whole system. Some of these factors can be interpreted in terms of well known common sources and/or transport/neutralization/deposition processes for the Antarctic plateau, but one of them stands out as quite novel.

In fact, factor 1 shows significant contributions of Cl^−^, Ca^2+^, Na^+^ and Mg^2+^ (listed in decreasing order of importance according to their factor loadings, ranging between 0.91 and 0.70), all positively correlated but not significantly correlated with ^10^Be, as already hinted at by Fig. 1. Factor 1 can be associated with primary aerosol inputs, namely the marine source (represented by Na^+^, Cl^−^, Mg^2+^ and a minor but still significant fraction of Ca^2+^) and crustal inputs (represented by the largest part of Ca^2+^). Both the inputs (primary marine and mineral dust) likely grouped in this factor show large variations in intensity and/or efficiency of transport processes (meridional and zonal wind strength) in this period, spanning the AIM events 9–11, as also visible from the single component behaviour in Fig. 1. This factor accounts for a large part of the explained variance (33%), in perfect agreement with what was observed at Dome C in the study of single sea salt markers^54^ although no statistical source apportionment had been accomplished so far.

The high factor loading of Ca^2+^ can be explained by its “double” source: crustal and marine, likely behaving similarly in this period. Although the association with typical marine components may sound surprising in the glacial period, it is noteworthy that a significant amount, although not the most part, of Ca^2+^ can be ascribed to sea salt, even in cold periods (e.g. 54). Indeed, calculating the fraction of sea salt Ca^2+^ (i.e. Ca^2+^ arising directly from sea spray) to the total budget of this component in the considered period, by using the Ca^2+^/Na^+^ ratio in sea water and Na^+^/Ca^2+^ ratio in the upper crust^55^, we obtain an average contribution of 26.2%.

**Figure 2:**
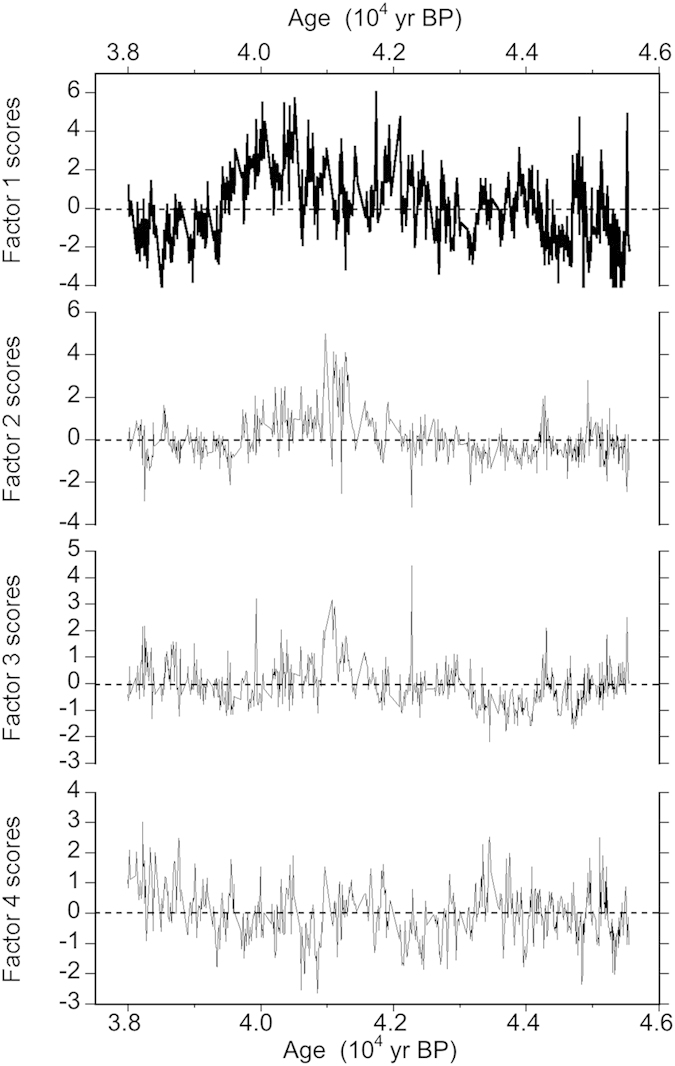
Temporal trend of Factor scores for Factors 1–4 produced by PCA.

The temporal variability of Factor 1 scores (shown in Fig. 2) shows a wavy pattern at millennial scale with positive values up to 6 around 42 kyr BP and in the 40.5–40 kyr BP dropping instead to very low, even negative values around 41 kyr BP, i.e. the time when the Laschamp event is centred. As also indicated by the extent of explained variance of this Factor, the sources here represented (primary marine and crustal inputs), control the aerosol chemistry budget along the examined period, except for the period before 43 kyr BP, after 39.5 kyr BP and at the maximum of the Laschamp event.

Factor 3 is represented almost uniquely by sulphate and it is probably driven mainly by volcanic and long-range inputs. Only two major independent sources affect sulphate concentration at Dome C, since primary marine source and crustal inputs can be neglected at this site for this marker^56^,^57^. The background level of sulphate in the Antarctic plateau is mostly controlled by the marine biogenic source^56^, while sulphate peaks are usually related to volcanic emissions, whose variability is stronger in intensity than marine aerosol delivery to the Antarctic plateau.

On the other hand, the particular relevance of sulphate records from plateau areas as providers of volcanic signatures is well known and was broadly studied and exploited at Dome C, for the reconstruction of volcanic eruptions and SO_2_ emission fluxes^58^, for dating purposes^59^,^60^ and for studying the volcanism-climate relationship^61^.

This is consistent with the separation of sulphate from MSA, that is present in this factor only with a very low negative coefficient and that, instead, accounts for Factor 4 almost by itself (factor loading of 0.92). This factor can be associated with the secondary marine source (biogenic productivity) and the lack of statistical match with sulphate indeed confirms that the long claimed interpretation of MSA and nssSO_4_^2−^ as “companion” representatives of marine biogenic source, already challenged by Preunkert *et al.*^57^ for Dome C in particular, needs a much better understanding.

In this factor, also Na^+^ and Mg^2+^ appear with relatively low factor loadings (0.40 and 0.39, respectively), possibly due to a not ideal separation of primary and secondary marine sources and/or to neutralization processes between methanesulfonic acid and sea salt particles, similarly to what was mentioned for calcium and nitrate.

The novelty of the PCA results is represented by Factor 2, explaining 17.0% of the variance of the system. This factor exhibits the contemporaneous presence of ^10^Be and nitrate with high factor loadings (0.74 and 0.82, respectively), explaining most of the variance of both components.

As shown in Fig. 2, the scores of Factor 2 are mostly negative before 42.5 kyr BP and after 39.5 kyr BP but are constantly positive in the 39.5–42.5 kyr BP time period, reaching high values (up to about 5) around the time of the maximum of the Laschamp event, pinpointing the importance of this new source to the snow depositions occurred during this period.

Given the well known cosmogenic origin of ^10^Be nuclides in the atmosphere and the contemporaneous presence in the same factor of nitrate, whose multiple sources in Antarctica are not fully understood but include stratospheric inputs, Factor 2 can be ascribed to the same original cosmogenic source: a larger GCR flow enhancing the production of cosmogenic isotopes and nitrate precursors (NOx).

Here the common variability of ^10^Be and nitrate is observed for the first time in the same ice core, during a major event of geomagnetic field excursion.

Ca^2+^ appears in Factor 2 with a low factor loading (0.24). Due to the completely different source of this component with respect to the other chemical components present in the same factor, this common variability (although accounting for a small fraction of Ca^2+^ total variability) can be explained by common deposition processes.

Indeed, analogously to other cosmogenic isotopes (such as ^7^Be and ^26^Al), ^10^Be nuclides are “particle active”, i.e. being able to react with compounds in the atmosphere, mainly forming oxides and adhering to/being dissolved into atmospheric solid/liquid-phase aerosol^61^. Thus, it is likely that the contemporaneous presence in this factor reflects a common deposition of sea salt, crustal particles and ^10^Be nuclides. Moreover, such a minor contribution of Ca^2+^ to this factor (mainly represented by ^10^Be and nitrate) can also be explained by other processes affecting calcium and nitrate, namely the “nitrate fixation” exerted by calcium carbonates on nitric acid occurring especially in the glacial period^63^.

**Figure 3:**
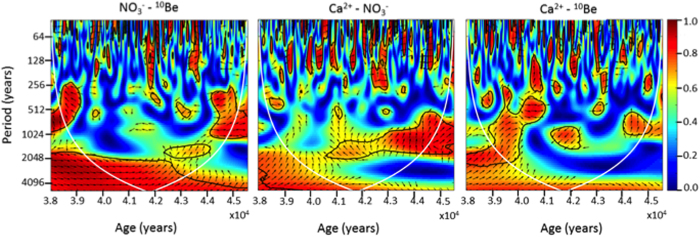
Wavelet coherence for nitrate and ^10^Be (**a**), nitrate and calcium (**b**) and ^10^Be and calcium (**c**) flux measured along the EDC core during the 38–45.5 kyr BP period. The colors indicate the coherence value (see the colorbar), the arrows point to the relative phase between the series (right-pointing arrows correspond to 0 degrees, up-pointing arrows –90 degrees, etc.). The areas of the 5% significance are bounded with black contours. The cone of influence, i.e., the areas of the edge effect, is shown with white lines.

In order to better appreciate and quantify the agreement between ^10^Be and NO_3_^−^ in the period containing the Laschamp event, a wavelet coherence analysis was applied whose results are shown in Fig. 3.

^10^Be and NO_3_^−^ (Fig. 3) show a strong and in-phase coherence at >2000 yr scale through the whole considered time period. Such an agreement, with fairly constant and high coherence values and in-phase relationship all along the investigated interval of time is not shown by any other pair of parameters and confirms the ^10^Be-NO_3_^−^ association highlighted by PCA analysis.

As previously mentioned, one of the main potential hindrances in using nitrate records from EDC, is the possible fixation effect exerted by Ca^2+^ during glacial periods (with aerosol rich in dust), possibly misleading the interpretation of nitrate enhancements during these periods. Quite surprisingly, calcium does not appear to share a significant variability with nitrate (Fig. 3) since only the area between 43 and 44.5 kyr BP shows a high coherence at 1000–2000 yr periods. Most importantly, the phase shift in this area is around 90°, which means a lag of 250–500 yr, making it difficult to find a physical explanation for this coherence.

The lack of a significant coherence over the investigated time period between Ca^2+^ and NO_3_^−^ has to be explored since nitrate shows considerably higher concentrations in glacial periods, and in particular when non-sea salt Ca^2+^ (nssCa^2+^) is high^54^,^63^.

It has been shown that the first order control on nitrate concentrations in central Antarctic ice cores can be ascribed to the dust content in ice (here represented by Ca^2+^): in periods characterised by high nssCa^2+^ concentration, nitrate appears to be at least partially preserved^63^, unlike periods with a low dust content (such as the Holocene) where a marked loss of nitrate from the snow layers occurs through a combination of sublimation and photolysis^27^.

Although here we investigate a glacial period and Ca^2+^ concentrations are higher than interglacials, it appears from our analysis (lack of significant coherence between nitrate and calcium and only a small amount of Ca^2+^ variability explained by Factor 2) that calcium is not the main factor in preserving nitrate or at least, that calcium content is not related to nitrate variability.

Following the approach adopted by Wolff *et al.*^54^, we plotted the nitrate concentrations against nssCa^2+^ concentrations during the period corresponding to the Laschamp event, only for nssCa^2+^ concentrations higher than 10 ppb, in order to identify a possible mathematical relationship of the “neutralization/fixation” effect induced by dust on nitrate during glacials. Unlike Wolff *et al.*^54^, who used the whole NO_3_^−^ and nssCa^2+^ from EDC ice core and achieved a significant linear correlation allowing to calculate the “excess NO_3_^−^” and to correlate them with the accumulation rate during the interglacials, we cannot observe any significant relationship in the period spanning the Laschamp event.

**Figure 4:**
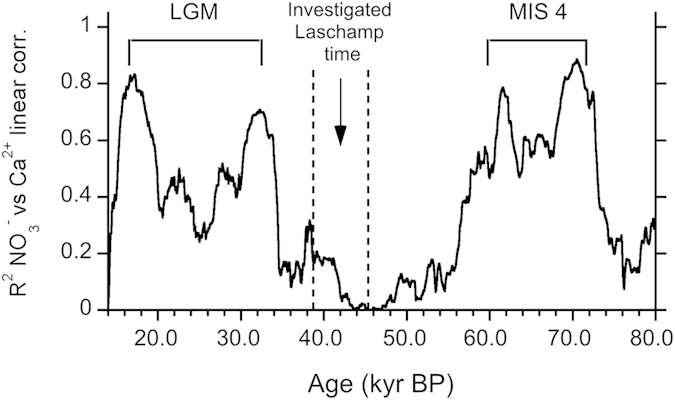
R^2^ of the running linear correlation (5-kyr window) between nitrate and calcium flux in the last glacial period (20–80 kyr BP).

In Fig. 4 we have reported the R^2^ of the running linear correlation between nitrate and calcium flux in the last glacial period (20–80 kyr BP).

A good linear correlation (R^2^ = 0.79, which implies 79% of common variability) between nitrate and calcium flux is found when the data set from the last glacial period is considered (20–80 kyr BP, see also Fig. 4), consistently with what found by Wolff *et al.*^54^ although in that case only calcium levels over a certain threshold (and thus considered able to fix nitrate efficiently) were taken into account.

Instead, when considering the period covering the Laschamp excursion, the data are much more dispersed and linear correlation coefficient markedly decreases, keeping always below 0.4 in the 38–45 kyr BP and dropping to 0.2 at 41 kyr BP, when the Laschamp event peaks.

On the contrary, excellent linear correlations are found during the coldest periods of the last glacial time (LGM – Last Glacial Maximum, about 17–32 kyr BP, and MIS 4 – Marine Isotope Stage 4, about 60- 70 kyr BP), when much larger dust amounts were supplied to the Antarctic plateau atmosphere^64^,^65^. During these periods, there is indeed a strong NO_3_^−^ and Ca^2+^ co-variability and dust delivered-Ca^2+^ can be considered as the driver of nitrate variability.

On the contrary, a “climatic” driver cannot be invoked for the nitrate peak occurred in time spanning the Laschamp event, for which we propose here an alternative explanation.

As can be observed from Fig. 3, ^10^Be shows a high coherence with Ca^2+^, with a 500–2000 yr period, in the 39.5–40.2 kyr BP time frame, thus not centred on the maximum of the Laschamp event, but rather on its latest part. The in-phase relationship exhibited only in the latest phase of the Laschamp can be explained by the contemporaneous occurrence of a large amount of dust delivered to the Dome C site and a still conspicuous ^10^Be atmospheric concentration, probably leading to a concomitant large dry deposition of both these species.

Hence, the data shown in Fig. 1 and mostly the results of a PCA and a wavelet analysis, suggest that the co-deposition effect for Ca^2+^ and ^10^Be and the fixation process for Ca^2+^ and nitrate appear not to be the main drivers of the common variability of ^10^Be and nitrate at Dome C, at least in the period covering the Laschamp event.

The direct comparison of a widely used proxy of GCR flux, ^10^Be, with other chemical markers measured along the same ice core, at the time of the Laschamp event, a major geomagnetic field excursion, allowed highlighting a significant common variability of ^10^Be with nitrate, which is ascribed to a common production source as well as to common transport and deposition processes on the central Antarctic ice sheet. The results obtained here hint at the potential of NO_3_^−^ to provide insights into major variations of cosmic ray flux, in this case likely caused by a significant dip in the geomagnetic field. This strengthens the case for NO_3_^−^ in polar ice also as a tracer of past solar activity. However, given the long time-scale considered here, it does not have any bearing on nitrate from polar ice as a tracer of short solar events such as those associated with solar flares or coronal mass ejections. Given the complex post-depositional processes affecting nitrate in the inner plateau, mostly controlled by local accumulation rate and atmospheric dust content, which have so far prevented an effective use of nitrate stratigraphies from the EDC ice core for paleoclimatic studies, the result obtained here appears to be relevant *per se* as well as offering a chance for EDC nitrate records to come into play again for specific paleoclimatic reconstruction.

## Additional Information

**How to cite this article**: Traversi, R. *et al.* The Laschamp geomagnetic excursion featured in nitrate record from EPICA-Dome C ice core. *Sci. Rep.* **6**, 20235; doi: 10.1038/srep20235 (2016).

## Figures and Tables

**Table 1:**
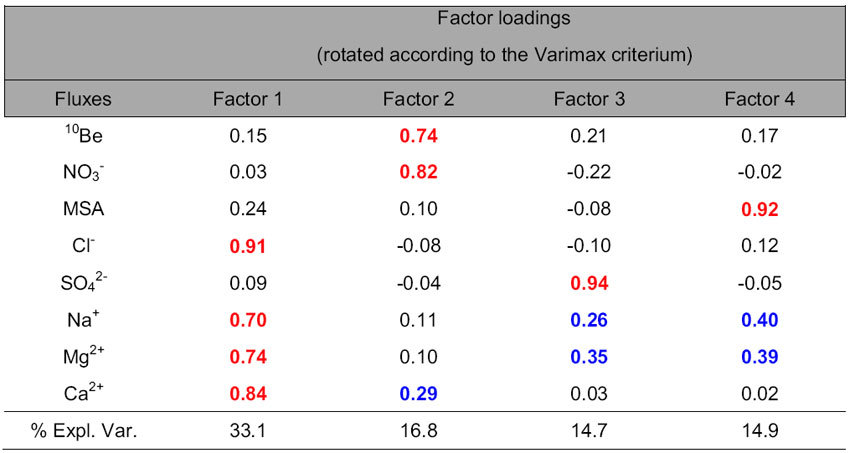
Factor loadings and fraction of total variance for PCA applied to ^10^Be and chemical markers fluxes during the Laschamp event from EDC ice core.

Factor loadings higher than 0.70 are highlighted in bold red and loadings lower than 0.70 but higher than 0.25 are highlighted in bold blue.
